# The Relationship Between the Pan-Immune–Inflammation Value (PIV) and Mortality in Elderly Critically Ill Patients with Sepsis: A Single-Centre Retrospective Study

**DOI:** 10.3390/jcm15103801

**Published:** 2026-05-14

**Authors:** Yeşim Şerife Bayraktar, Hasan Gazi Uyar, Yasemin Cebeci, Hasan Özkaya, Jale Bengi Çelik

**Affiliations:** 1 Division of Intensive Care, Department of Anesthesiology and Reanimation, Faculty of Medicine, Selcuk University, Konya 42130, Turkey; drhozk42@outlook.com (H.Ö.); jalecelik@hotmail.com (J.B.Ç.); 2 Division of Intensive Care, Department of Emergency Medicine, Faculty of Medicine, Selcuk University, Konya 42130, Turkey; dr.hasangaziuyar@gmail.com; 3 Division of Intensive Care, Department of Anesthesiology and Reanimation, Beyhekim Training and Research Hospital, Konya 42060, Turkey; dryasemin.cebeci@gmail.com

**Keywords:** sepsis, pan-immune–inflammation score, geriatrics, intensive care unit, mortality, immunosenescence

## Abstract

**Background**: The pan-immune–inflammation (PIV) score is a hematological index derived from neutrophil, platelet, monocyte and lymphocyte counts. It has been demonstrated that it has high prognostic value in oncological patients. The aim of this study was to evaluate the association between PIV and 28-day mortality in elderly (≥65 years) critically ill patients admitted to the intensive care unit (ICU) with a diagnosis of sepsis. **Methods**: This single-centre retrospective study included 96 patients aged ≥65 years who were admitted to the ICU with a diagnosis of sepsis according to the Sepsis-3 criteria between 15 July 2024 and 15 July 2025. Patients were divided into low- and high-PIV groups based on the median PIV. Cox proportional hazards regression analysis and Kaplan–Meier survival analysis were performed. **Results**: The overall 28-day mortality rate was found to be 55.2% (*n* = 53). The median PIV was 866.58 (IQR: 497.34–1978.43). The PIV was shown not to be a significant predictor of 28-day mortality (AUC: 0.550; *p* = 0.400). No difference in survival was observed between the low- and high-PIV groups in the Kaplan–Meier analysis (log-rank *p* = 0.662). In multivariate Cox regression, high creatinine (HR: 2.683; *p* < 0.001), high calcium (HR: 2.312; *p* = 0.004), a low partial thromboplastin time (HR: 0.396; *p* = 0.005) and a requirement for vasopressors (HR: 2.225; *p* = 0.025) were identified as independent predictors of mortality. In the Kaplan–Meier analysis for 28-day survival, chronic obstructive pulmonary disease (*p* = 0.023) and chronic renal disease (*p* = 0.034) were found to be significantly associated with poorer survival. **Conclusions**: The PIV is unable to predict 28-day mortality in elderly critically ill patients diagnosed with sepsis. This finding suggests that immunosenescence and inflammaging reduce the predictive power of composite hematological indices. Markers of organ dysfunction, coagulopathy and hemodynamic instability remain more reliable prognostic indicators in geriatric patients with sepsis.

## 1. Introduction

Sepsis is defined as life-threatening organ dysfunction resulting from a dysregulated immune response to infection [1]. Currently, it is both a leading cause of death in intensive care units (ICUs) and a public health issue [1,2,3]. The incidence and severity of sepsis increase significantly with advancing age [4]. The majority of sepsis cases occur in patients aged 65 and over, and this proportion is expected to rise further as the elderly population grows [5]. Immunosenescence and inflammaging, which are associated with the ageing process, complicate the pathophysiological course of sepsis in the geriatric population [3]. Chronic inflammation associated with ageing is referred to as ‘inflammaging’ [6]. Cases of sepsis are on the rise due to an ageing population whose immune systems have weakened as a result of the natural ageing process [7]. On the other hand, immunosenescence is a sequence of age-related changes that impact the immune system and result in an increased susceptibility to infectious diseases over time [8]. The reduction in the antigen-specific adaptive immune response is the most noticeable aspect. T-cell depletion in sepsis in the elderly increases hospital-acquired infections after septicemia and may potentially result in death during the subacute period [6].

In cases of sepsis, early diagnosis and accurate prognostic assessment are of critical importance in the management of clinical decisions regarding early treatment, the appropriate use of resources, and care objectives. Established severity scoring systems such as the Acute Physiology and Chronic Health Evaluation II (APACHE II) and the Sequential Organ Failure Assessment (SOFA) scores are used as standard prognostic tools [5]. In addition, the literature has shown that factors such as polypharmacy, comorbidities and functional status in older patients have a greater impact on long-term outcomes than age alone [5,9]. In addition to routine scoring systems, there is a need for new, low-cost, and easily accessible biomarkers.

Markers derived from routine complete blood count parameters are used for prognostic purposes in many clinical conditions. Numerous studies have shown that clinical outcomes in sepsis, cardiovascular disease, and malignancy are correlated with markers such as the neutrophil–lymphocyte ratio (NLR), the systemic immune–inflammation index (SII), and the platelet–lymphocyte ratio [10,11]. In recent years, the pan-immune–inflammation value (PIV) has been proposed as a more comprehensive indicator of systemic inflammation and immune status, as it incorporates the four key components of the innate and adaptive immune response: neutrophils, platelets, monocytes and lymphocytes [12].

The PIV was originally developed for the oncology patient population. Typically, prognostic scores are calculated by dividing the counts of pro-inflammatory cells—such as neutrophils, platelets, and monocytes—by the count of lymphocytes, which are the primary generators of anti-cancer immunity in the tumour microenvironment [13,14]. It has been shown that high PIVs are consistently correlated with poor survival in many types of cancer [12,14,15]. Prognostic value has been reported in myocardial infarction, hypertension and vasculitis [16,17]. However, the prognostic value of these findings in non-oncological critical care patients, particularly elderly patients with sepsis, remains unclear.

The primary aim was to determine the ability of the PIV to predict 28-day mortality in elderly patients with sepsis admitted to the intensive care unit (ICU). Secondary aims included identifying independent clinical and laboratory parameters as predictors of mortality.

## 2. Materials and Methods

### 2.1. Study Design and Setting

This study is a retrospective, single-centre study conducted in the intensive care unit (ICU) of the Department of Anesthesiology and Resuscitation at Selçuk University. Approval was obtained from the Ethics Committee (Selcuk University Faculty of Medicine Ethics Committee, Konya, Turkey, approval number: 2025/451; date: 29 July 2025). The study protocol complies with the Helsinki Declaration.

### 2.2. Participants

All patients aged 65 years or older admitted to the ICU with a diagnosis of sepsis between 15 July 2024 and 15 July 2025 were screened. Sepsis was defined as an increase of ≥2 points in the SOFA score in the presence of a suspected or confirmed infection, in accordance with the Third International Consensus Definitions (Sepsis-3) [1].

#### 2.2.1. Inclusion Criteria

Patients admitted to the ICU who were initially diagnosed with sepsis according to the Surviving Sepsis Campaign criteria, aged ≥65 years, and for whom complete data on neutrophil, platelet, monocyte or lymphocyte counts were available within 24 h of admission to the ICU were included in this study.

#### 2.2.2. Exclusion Criteria

Patients who were missing data on neutrophil, platelet, monocyte or lymphocyte counts within the first 24 h of admission to the ICU; those under 65 years of age; those with a known diagnosis of malignancy; neutropenic patients; and patients diagnosed with human immunodeficiency virus infection were excluded from this study.

**Figure 1:**
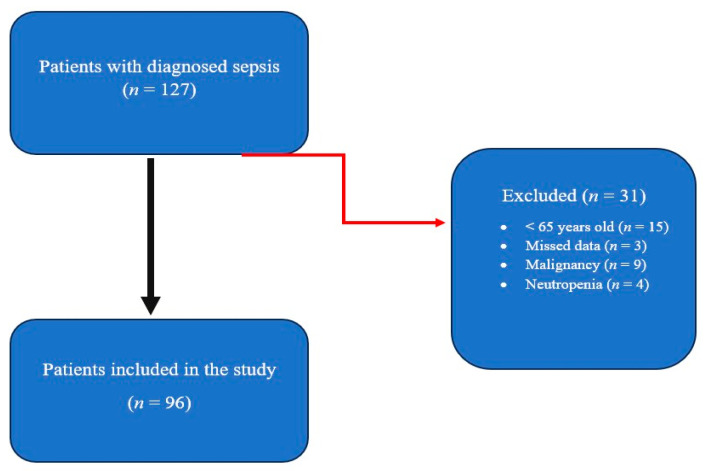
Patient selection flowchart.

Patients who were diagnosed with sepsis and received monitoring and treatment in the ICU on the dates identified by the hospital’s records system were screened. Of the 127 patients diagnosed with sepsis, 15 were under the age of 65; 3 were excluded due to missing data, 4 due to neutropenia, and 9 due to malignancy (Figure 1). A total of 96 patients were included in this study.

This study was conducted in accordance with the STROBE statement for observational studies.

### 2.3. Data Collection and Definitions

Demographic, clinical and laboratory data were obtained from the hospital’s electronic medical records. The variables collected included age, gender, GCS, SOFA score, APACHE II score, intubation status on admission, and comorbidities, namely hypertension, cerebrovascular accident (CVA), chronic obstructive pulmonary disease (COPD), cardiovascular disease (CVD), and chronic renal disease (CRD).

Laboratory parameters were obtained within the first 24 h of admission to the ICU: arterial blood gas analysis, complete blood count, aspartate aminotransferase (AST), alanine aminotransferase (ALT), urea, creatinine, sodium (Na), calcium (Ca), C-reactive protein (CRP), potassium, albumin, procalcitonin, prothrombin time (PT), partial thromboplastin time (PTT), and INR.

Additional serum biochemical parameters were measured using the Roche cobas c701 and c702 modular analyzers (Roche Diagnostics, Mannheim, Germany) with the manufacturer’s proprietary reagent kits as follows: AST acc. to the IFCC kit (Cat. No: 05850819190), ALT acc. to the IFCC kit (Cat. No: 05850797190), urea/BUN kit (Cat. No: 05171873190), creatinine acc. to the plus ver.2 (enzymatic) kit (Cat. No: 05168589190), and calcium acc. to the Gen.2 kit (Cat. No: 05168449190). Serum sodium and potassium concentrations were determined potentiometrically using the cobas 8000 ISE module (ion-selective electrode; Roche Diagnostics, Mannheim, Germany). Serum CRP levels were quantified by particle-enhanced immunonephelometry using the BN II System (Siemens Healthcare Diagnostics Products GmbH, Marburg, Germany) with the CardioPhase hsCRP reagent (Ref. No: OQIY21). Procalcitonin levels were measured using the Elecsys BRAHMS PCT electrochemiluminescence immunoassay (ECLIA) on the cobas e 801 analytical unit (Roche Diagnostics, Mannheim, Germany; Cat. No: 08828679190). The PIV was calculated using the following formula: PIV = (neutrophil count × platelet count × monocyte count)/lymphocyte count; all cell counts are expressed in 10^3^/µL [12]. The NLR and SII were also calculated. The neutrophil-to-lymphocyte ratio (NLR) was calculated as follows: neutrophil count (10^3^/µL)/lymphocyte count (10^3^/µL) [12]. The systemic immune–inflammation index (SII) was defined as (neutrophil count × platelet count)/lymphocyte count [18].

### 2.4. Primary and Secondary Outcomes

The primary outcome of this study was 28-day all-cause ICU mortality. The secondary outcomes were length of ICU stay and vasopressor requirement.

### 2.5. Statistical Analysis

All statistical analyses were performed using R 4.2.1 (https://www.r-project.org/, accessed on 16 March 2026). The normality of continuous variables was assessed using the Shapiro–Wilk test and Q–Q plots, while homogeneity of variances was evaluated using Levene’s test. Statistical test selection was based on data distribution and homogeneity of variance. For group comparisons, the independent samples *t*-test was used for normally distributed variables with equal variances, whereas Welch’s *t*-test was applied when variances were unequal. The Mann–Whitney U test was used for non-normally distributed variables. Categorical variables were analyzed using Pearson’s chi-square test, and Yates’ continuity correction was applied when expected cell counts were low. The study population was divided into two groups according to the median pan-immune–inflammation value (PIV) as the cut-off point. Univariate Cox proportional hazards regression analysis was performed to identify potential prognostic factors, and variables found to be significant in univariate analysis were subsequently entered into a multivariate Cox proportional hazards regression model using a stepwise (backward likelihood ratio) selection method. Hazard ratios (HRs) were reported with 95% confidence intervals. Receiver operating characteristic (ROC) curve analysis was used to evaluate the predictive performance of clinical and laboratory parameters for 28-day ICU mortality. The area under the curve (AUC) was calculated with 95% confidence intervals. Optimal cut-off values were determined using the Youden index, and sensitivity, specificity, positive predictive value, and negative predictive value were calculated with 95% confidence intervals. Overall survival was analyzed using the Kaplan–Meier method, and differences between groups were assessed using the log-rank test. Survival times were reported as median values with 95% confidence intervals, and 28-day survival probabilities were calculated for each group. A *p*-value < 0.05 was considered statistically significant.

## 3. Results

### 3.1. Baseline Characteristics

Descriptive data on the patients’ clinical and laboratory parameters are presented in Table 1. The patients’ average age was 74.74 ± 8.46 years; 47.9% (*n* = 46) were male and 52.1% (*n* = 50) were female. The median APACHE, GCS, and SOFA scores were 18.00 (9.50–23.50), 14.00 (12.00–15.00), and 3.00 (2.00–4.00), respectively. At admission, 43.8% of patients were intubated. The most common comorbidities were hypertension, COPD, cerebrovascular disease, cardiovascular disease, and chronic kidney disease. Overall, 64.6% of patients required vasopressor support. The median ICU stay was 15.00 (6.75–31.00) days, and the 28-day mortality rate was 55.2% (Table 1).

### 3.2. Comparison of Low- and High-PIV Groups

**Figure 2:**
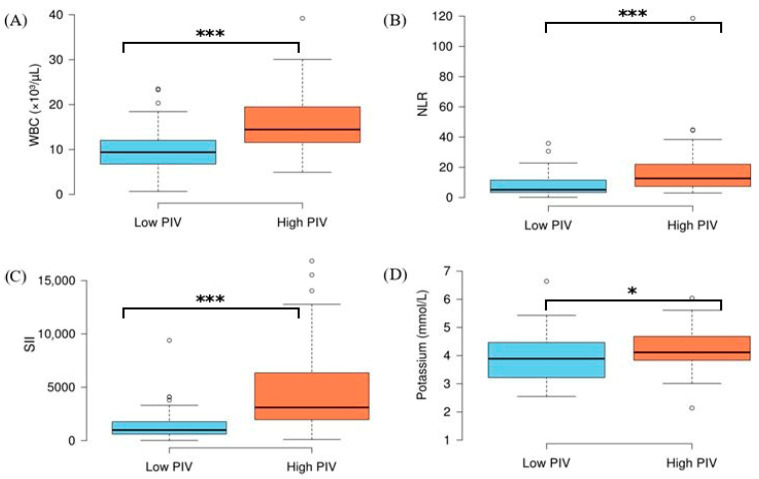
WBC (**A**), NLR (**B**), SII (**C**), and potassium (**D**) levels in intensive care patients stratified by low and high PIVs. Horizontal lines in each box indicate median. * *p* < 0.05 and *** *p* < 0.001 (independent samples *t*-test for WBC and potassium; Mann–Whitney U test for NLR and SII).

A comparison of the low- and high-PIV groups based on clinical and laboratory findings is presented in Table 2. In the high-PIV group, the prevalence of hypertension (HT) was found to be higher than that in the low-PIV group, whilst the prevalence of CRD was lower. Furthermore, white blood cell (WBC) count, the neutrophil-to-lymphocyte ratio (NLR), the serum inflammatory index (SII) and potassium levels were found to be significantly higher in patients in the high-PIV group compared to the low-PIV group (Figure 2). No significant differences were observed between the low- and high-PIV groups in other clinical and laboratory parameters (all *p* > 0.05).

**Figure 3:**
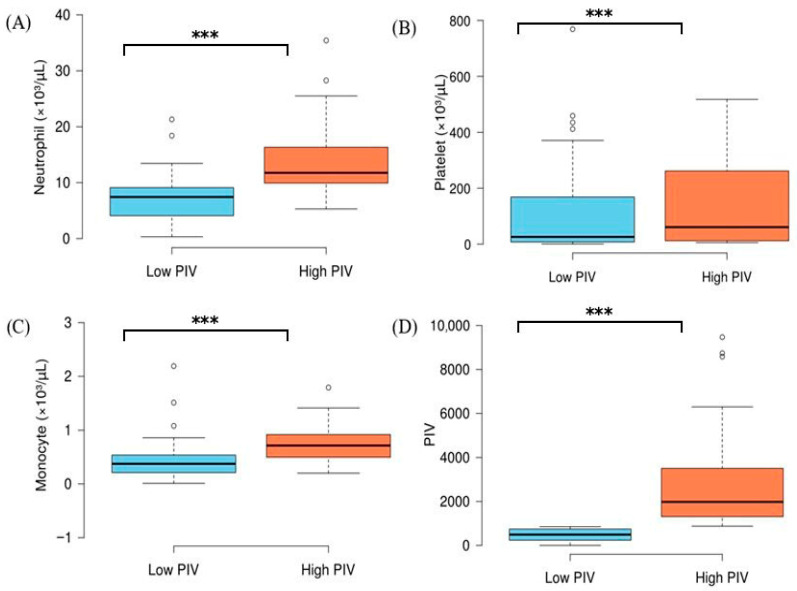
Neutrophil (**A**), platelet (**B**), monocyte (**C**), and PIV (**D**) levels in intensive care patients stratified by low and high PIVs. Horizontal lines in each box indicate median. *** *p* < 0.001 (Mann–Whitney U test).

The comparison of complete blood count parameters between low- and high-PIV groups is presented in Table 3. Neutrophil, platelet, and monocyte counts, as well as PIV levels, were significantly higher in the high-PIV group, while no significant difference was observed in lymphocyte levels between the groups (*p* > 0.05) (Figure 3).

### 3.3. Predictive Performance of Laboratory Parameters

**Figure 4:**
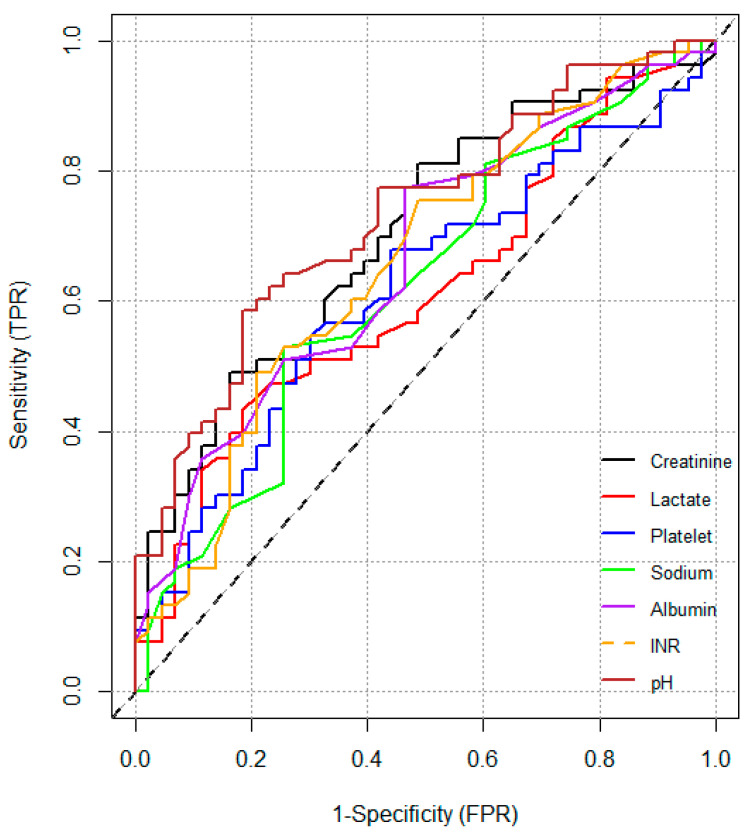
ROC curves of laboratory parameters and vasopressor requirement for mortality in intensive care patients.

The cut-off values and diagnostic performance of laboratory parameters predictive of mortality in the ICU are presented in Table 4. The highest AUC was 0.731 (95% CI: 0.631–0.831) for pH. This was followed by creatinine at 0.703 (95% CI: 0.598–0.807), albumin at 0.665 (95% CI: 0.556–0.774), INR at 0.660 (95% CI: 0.549–0.770), sodium at 0.622 (0.509–0.735), platelets at 0.618 (95% CI: 0.505–0.731) and lactate at 0.614 (95% CI: 0.500–0.727) (Figure 4). Other biochemical parameters were not found to be statistically significant in predicting mortality.

### 3.4. Cox Proportional Hazards Analysis

In univariate Cox regression analysis (Model 1), elevated lactate, ALT, AST, creatinine, calcium, procalcitonin, INR, and PT levels were associated with increased mortality, whereas higher platelet counts and sodium and albumin levels were protective. Additionally, COPD, chronic kidney disease, and vasopressor requirement were associated with higher mortality risk. In the age- and sex-adjusted model (Model 2), age and calcium remained significant risk factors, while higher albumin and PTT levels were associated with reduced mortality. In multivariate Cox regression analysis (Model 3), only elevated creatinine and calcium levels, increased vasopressor requirement, and low PTT remained independent predictors of mortality (Table 5).

### 3.5. Survival Analysis

**Figure 5:**
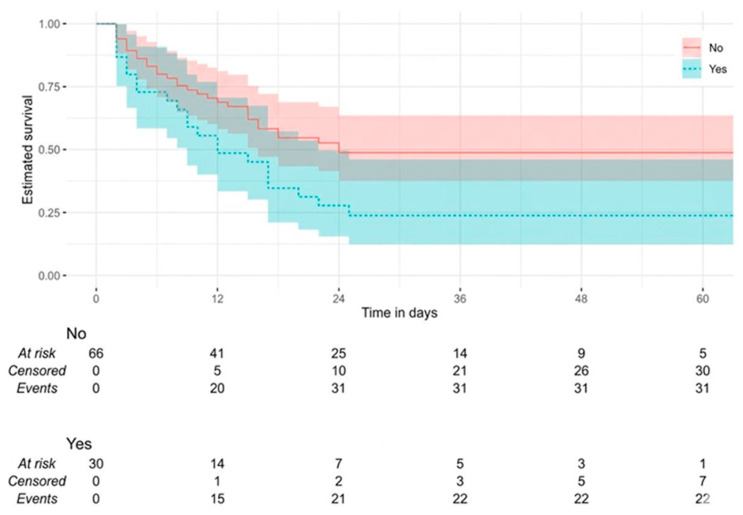
Kaplan–Meier survival curves for overall survival stratified by chronic obstructive pulmonary disease status (present vs. absent).

**Figure 6:**
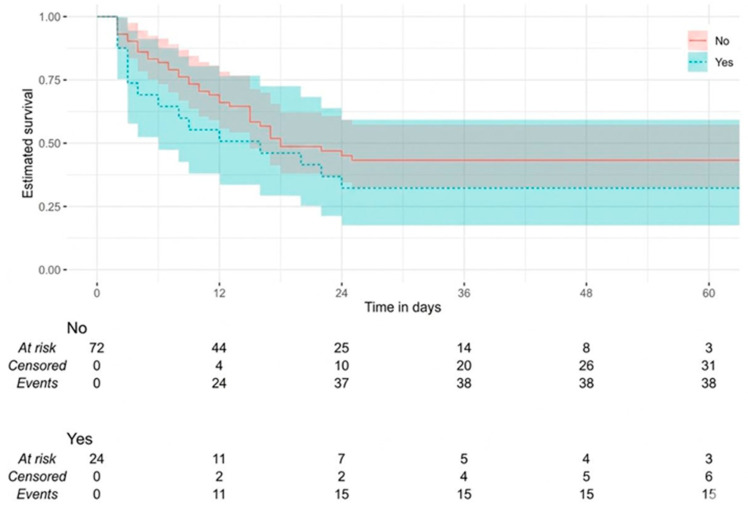
Kaplan–Meier survival curves for overall survival stratified by chronic renal disease status (present vs. absent).

The 28-day overall survival (OS) results for intensive care patients are presented in Table 6. The median survival time for all patients was found to be 18.00 days (95% CI: 15.00–25.00). The median survival values for the low-PIV group (18.00 days [95% CI: 9.00–25.00]) and the high-PIV group (18.00 days [95% CI: 13.00–24.00]) were found to be equivalent. However, when 28-day survival probabilities were examined using Kaplan–Meier analysis, the median survival time for patients with COPD (12.00 days [95% CI: 7.00–22.00]) was found to be lower than that of patients without COPD (24.00 days [95% CI: 15.00–24.00]) (*p* = 0.023) (Figure 5). Furthermore, the median survival time for patients with CRD (11.00 days [95% CI: 5.00–24.00]) was significantly lower (*p* = 0.034) (Figure 6). No statistically significant differences were observed in OS distributions with respect to age, sex and other comorbidities (all *p* > 0.05).

## 4. Discussion

In our study, we found that the PIV measured on admission to the ICU in elderly critically ill patients diagnosed with sepsis did not predict 28-day mortality, contrary to previous studies in the literature [19,20]. The PIV showed a low AUC (0.550) in the ROC analysis, and the Kaplan–Meier analysis confirmed that there was no difference in survival between the low- and high-PIV groups. Instead, high creatinine, high calcium, low PTT and vasopressor requirement were found to be independent predictors in the multivariate Cox regression analysis. To the best of our knowledge, our study is one of the first to investigate the prognostic value of the PIV in geriatric sepsis patients.

There are studies in the literature supporting the prognostic value of the PIV in cancer patients [12,14,15]. Our findings are consistent with the failure of the PIV to predict mortality in non-oncological settings. In cancer patients, the balance between pro-inflammatory and anti-tumour immune cells is directly linked to tumour progression. In sepsis, however, there is a rapidly developing dysregulated immune response. Rapid shifts between hyperinflammation and immunosuppression can occur within hours. A single PIV measurement taken at the time of presentation may be insufficient to capture this rapidly changing immune profile [6,7].

Despite recent advances in treating sepsis, the mortality rate remains high. In various studies, this rate can vary between 10% and 70%, depending on the average age of participants, the severity of sepsis, the country in which the study was conducted, and the prevalence of comorbid conditions. In two prospective studies conducted on elderly patients with critical sepsis, Tekin et al. reported an overall mortality rate of 41% in 202 patients, whilst Martin-Loeches et al. reported a rate of 48.8% in 1490 patients [5,21]. In people aged 80 and over, the mortality rate rises to between 40% and 80% [3]. In our study, the 28-day mortality rate was found to be 55.2%. Although the mortality rate is slightly higher than that reported in the literature, the high prevalence of comorbidities among patients and the high requirement for vasopressors support the view that these are factors contributing to increased mortality. Vasopressor requirement reflects hemodynamic instability and progression to septic shock. In patients with septic shock, the expected mortality rate is high and has been associated with poor outcomes [1]. Our study also reveals a strong association between mortality and vasopressor requirement. This supports the finding that our geriatric sepsis patients progress to shock and have a high mortality rate.

In our study, the patient population had a high burden of comorbidity (36.5% hypertension, 31.3% COPD, 26.0% CVA, 25.0% cardiovascular disease, 19.8% CRD). This situation leads to confounding changes in blood cell counts. CRD may be associated with uremic thrombocytopenia and is linked to altered leukocyte profiles. COPD independently elevates neutrophil counts via chronic airway inflammation [22]. Hematological abnormalities associated with this comorbidity may be masking the prognostic significance of the PIV in geriatric patients.

In a study involving 82 patients with septic shock, the PIV predicted survival in univariate analysis but was not found to be significant in multivariate analysis [20]. A retrospective study involving 11,331 patients with sepsis identified a non-linear relationship between log2-PIV and mortality. The study reported that the PIV had strong prognostic value in the subgroup comprising patients aged ≥ 65 years [19]. The study was also conducted in all adult patients with sepsis aged 18 years and over. There was no age restriction. In the study, patients in the group with a higher Log2-PIV had a higher prevalence of comorbidities such as congestive heart failure, chronic lung disease and chronic kidney disease. Although our age range differs from that of the study, the Kaplan–Meier analysis conducted in our study found that COPD and CRD were significantly associated with poorer survival. These findings suggest that impaired respiratory and renal function in elderly patients with sepsis will increase the need for organ support systems and indirectly contribute to mortality.

Studies in the geriatric population have shown that this population is subject to immunosenescence, characterized by thymic involution, a reduction in the output of naive T cells, the accumulation of senescent memory cells, and impaired neutrophil/monocyte function [8]. In parallel, inflammaging creates a chronic, low-grade inflammatory environment that can elevate basal PIVs, regardless of the severity of the infection [23]. These processes, taken together, may reduce the discriminatory power of the PIV by narrowing the range of values between survivors and non-survivors in elderly populations. Indeed, a recent meta-analysis of the relationship between the SII and mortality in subgroup analyses demonstrated a significantly stronger association in patients under 67 years of age compared with those aged 67 and over (*p* = 0.04) [18]. As the SII (AUC: 0.594) includes three of the four components of the PIV, this finding supports the notion that hematological indices perform poorly in elderly populations. Similarly, the NLR (AUC: 0.507) is not significantly associated with mortality. This supports the view that all three markers have limited prognostic value in elderly patients with sepsis. This is likely due to hematological changes associated with immunosenescence and comorbidity. As shown in Table 3, neutrophil, platelet, and monocyte counts were significantly higher in the high-PIV group, whereas lymphocyte levels did not differ between groups. The preservation of lymphocyte counts across both PIV strata, despite marked elevations in pro-inflammatory cell lineages, may reflect the age-related contraction of the lymphocyte compartment driven by immunosenescence, which compresses the baseline range and limits its discriminatory capacity in elderly patients [8,23]. This finding further supports the notion that PIV-based risk stratification is attenuated in the geriatric population, as the lymphocyte denominator—a key driver of PIV variability—loses its discriminatory power in this age group.

The finding in our study that creatinine is the strongest independent predictor of mortality (HR: 2.683) is consistent with extensive studies linking acute kidney injury to sepsis mortality. In a large-scale retrospective study evaluating elderly critically ill patients with sepsis-associated acute kidney injury, a serum creatinine level of ≥1.5 mg/dL was shown to be an independent risk factor for 28-day mortality (HR: 1.216; 95% CI: 1.037–1.427) [24]. In another study involving 1752 patients with geriatric sepsis, it was reported that a serum creatinine level >200 μmol/L was a significant predictor of mortality [25].

In our study, elevated calcium—an independent predictor—may reflect critical disease-related disturbances in calcium homeostasis, including impaired parathyroid function and vitamin D deficiency, which are frequently observed in elderly patients with sepsis [26]. In a study encompassing 3016 septic patients, a U-shaped relationship was identified between serum calcium levels and 28-day mortality in patients with sepsis. Both hypocalcaemia and hypercalcaemia have been associated with an increased risk of mortality [27].

Our study found a strong association between low PTT and mortality. In a study of 647 patients with severe sepsis or septic shock, prolonged activated partial thromboplastin time and elevated PT-INR values observed at the time of admission to the ICU were associated with increased mortality. Although this finding is contrary to our study, it may reflect consumption coagulopathy in which the PTT may paradoxically shorten before coagulation factors are depleted [28]. It may serve as an early marker for undiagnosed disseminated intravascular coagulation (DIC). A shortened PTT may also reflect dehydration and hemoconcentration, which are frequently observed in elderly septic patients. This suggests that baseline PTT values in elderly patients with sepsis may carry different prognostic information beyond the classic prolonged PTT pattern seen in advanced consumption coagulopathy. Prospective studies incorporating serial coagulation measurements, thrombin generation assays and endothelial activation markers would be valuable to confirm the prognostic role of a shortened PTT in the geriatric sepsis population.

Our ROC analysis demonstrated that no individual laboratory parameter achieved an AUC exceeding 0.731, indicating that single biomarkers have limited clinical utility for mortality prediction in geriatric sepsis. This is consistent with the multifactorial pathophysiology of sepsis, in which organ dysfunction, hemodynamic instability, coagulopathy, and immune dysregulation interact simultaneously. Accordingly, composite scoring systems such as APACHE II and SOFA, which integrate multiple physiological variables, remain more appropriate for clinical prognostication. Future research should explore multi-biomarker panels or combined models incorporating both hematological indices and clinical severity scores to improve predictive performance in this population. In this context, Zhang et al. demonstrated that combining IL-8 with SOFA scores yielded superior predictive performance compared to any single index alone in elderly sepsis patients, further supporting the need for multi-parameter approaches [29].

This study has certain limitations. Its retrospective, single-centre design limits generalisability and carries the potential for selection bias. Our sample size is small (*n* = 96), which may have limited the statistical power to detect small differences. In this study, the PIV was measured only upon admission to the ICU. As sepsis is a dynamic process, serial measurements could provide additional information regarding prognosis. Although nutritional status was evaluated in terms of albumin and hypoproteinemia, there are many parameters of nutrition, and others were not assessed. The source of sepsis, including culture results, was not included. The median PIV of the patients was accepted as the threshold value; however, this value may not represent the optimal threshold for outcome prediction in geriatric patients. Furthermore, data on the use of corticosteroids and other concomitant immunomodulatory interventions were not systematically recorded. Given that corticosteroids may directly influence clinical outcomes in septic patients via the hematological profile used in the calculation of the PIV (e.g., neutrophilia, lymphopenia), their potential confounding effects on the predictive performance of the PIV cannot be disregarded and constitute a significant limitation. Similarly, formal frailty assessment tools (such as the Clinical Frailty Scale or Frailty Index) were not available in the electronic records and could therefore not be included in the analysis. As frailty is increasingly recognized as a key determinant of outcome in elderly critically ill patients, independent of age and comorbidity burden, its absence may have limited our ability to fully account for baseline frailty. Future prospective studies should aim to prospectively collect data on standardized frailty measurements alongside exposure to corticosteroids and other concurrent interventions, and conduct stratified analyses to clarify the independent prognostic contribution of the PIV in geriatric sepsis.

## 5. Conclusions

The PIV measured on admission to the ICU in elderly critically ill patients diagnosed with sepsis does not predict 28-day mortality. In geriatric patients, immunosenescence, inflammaging and hematological changes associated with comorbidity may reduce the predictive power of this index. In geriatric sepsis patients, traditional predictors of organ dysfunction (creatinine, calcium), coagulopathy (PTT) and hemodynamic instability (requirement for vasopressors) retain their reliability in determining prognosis. There is a need for large-sample-size, multicentre, prospective studies in geriatric intensive care patients that include serial PIV measurements and frailty assessments. Furthermore, extending the follow-up period beyond 28 days to include 3- and 6-month outcomes may provide a more comprehensive evaluation of the prognostic performance of the PIV and other hematological indices in this population, as longer-term mortality, readmission, and functional decline are particularly relevant endpoints in geriatric sepsis survivors. Dynamic assessment of the PIV through serial measurements during ICU stay could also help to capture the rapidly changing immune profile of sepsis and identify patterns of clinical response that a single admission value may miss.

## Figures and Tables

**Table 1:** Clinical and laboratory parameters.

Parameters	*n* (%), Median (IQR) or Mean ± SD
Age	74.74 ± 8.46
Age group	
65–74	53 (55.2)
75–84	26 (27.1)
≥85	17 (17.7)
Sex (M/F)	46 (47.9)/50 (52.1)
APACHE	18.00 (9.50–23.50)
Intubated on admission	42 (43.8)
GCS	14.00 (12.00–15.00)
SOFA	3.00 (2.00–4.00)
HT	35 (36.5)
CVA	25 (26.0)
COPD	30 (31.3)
Cardiovascular disease	24 (25.0)
Chronic renal disease	19 (19.8)
pH	7.40 (7.29–7.46)
pO_2_	89.00 (87.00–94.70)
pCO_2_	40.07 ± 8.91
Lactate	1.80 (1.40–3.10)
Hemoglobin	9.80 ± 2.07
WBC	12.92 ± 6.65
Neutrophil	9.48 (7.10–13.00)
Platelet	226.00 (147.75–320.00)
Monocyte	0.51 (0.31–0.77)
Lymphocyte	1.02 (0.64–1.54)
NLR	8.69 (4.80–17.04)
SII	1870.11 (957.13–3499.65)
PIV	866.58 (497.34–1978.43)
ALT	19.50 (10.00–42.50)
AST	33.00 (15.00–70.50)
Creatinine	1.48 (0.79–2.44)
Urea	83.00 (56.75–143.00)
Calcium	7.97 ± 0.88
Sodium	142.44 ± 7.58
Potassium	4.08 ± 0.80
Procalcitonin	1.90 (0.45–7.27)
CRP	125.50 (74.25–179.00)
Albumin	2.78 ± 0.61
INR	1.21 (1.08–1.47)
PT	14.25 (13.07–16.20)
PTT	30.93 ± 7.81
VP requirement	62 (64.6)
Length of ICU stay (days)	15.00 (6.75–31.00)
28-day mortality	53 (55.2)

SD = standard deviation. IQR = interquartile range. M: male. F: female.

**Table 2:** Comparison of clinical and laboratory parameters between low- and high-PIV groups.

Parameters	Groups		
	Low PIV (*n* = 48)	High PIV (*n* = 48)	*p*-Value
HT	12 (25.0)	23 (47.9)	**0.034** ^1^
Chronic Renal Disease	14 (29.2)	5 (10.4)	**0.040** ^1^
WBC	9.75 ± 4.95	16.09 ± 6.66	**<** **0.001** ^2^
NLR	5.16 (3.52–11.36)	12.70 (7.48–21.59)	**<** **0.001** ^3^
SII	989.63 (615.83–1726.82)	3107.31 (2007.30–6236.06)	**<** **0.001** ^3^
Potassium	3.91 ± 0.81	4.25 ± 0.76	**0.039** ^4^

Data are presented as the mean ± standard deviation or median (interquartile range, 25th–75th percentile) for numerical variables and are described as the count (*n*) and percentage (%) for categorical variables. Bold values indicate statistical significance (*p* < 0.05). ^1^ Chi-square test with Yates’ continuity correction. ^2^ Welch *t*-test. ^3^ Mann–Whitney U test. ^4^ Independent samples *t*-test.

**Table 3:** Comparison of complete blood count parameters between the low- and high-PIV groups.

Parameters	Groups		
	Low PIV (*n* = 48)	High PIV (*n* = 48)	*p*-Value *
Neutrophil	7.44 (4.12–9.12)	11.77 (9.98–16.20)	**<** **0.001**
Platelet	168.50 (106.75–271.75)	261.50 (203.00–362.50)	**<** **0.001**
Monocyte	0.38 (0.22–0.53)	0.71 (0.50–0.92)	**<** **0.001**
Lymphocyte	1.04 (0.68–1.54)	1.02 (0.63–1.55)	0.733
PIV	495.77 (253.49–741.73)	1982.86 (1313.11–3509.88)	**<** **0.001**

Data are presented as the median (interquartile range, 25th–75th percentile). Bold values indicate statistical significance (*p* < 0.05). * Mann–Whitney U test.

**Table 4:** ROC curve analysis and statistical diagnostic measures of various parameters for predicting 28-day ICU mortality in elderly patients with sepsis.

Parameters	ROC Curve Analysis			Statistical Diagnostic Measures (%)			
	AUC (95% CI)	*p*-Value	Cut-Off	Sensitivity	Specificity	PPV	NPV
Age	0.538 (0.421–0.654)	0.525	≥83.50				
APACHE	0.507 (0.387–0.626)	0.915	≥16.50				
GCS	0.523 (0.408–0.639)	0.693	≥15.50				
SOFA	0.565 (0.449–0.681)	0.272	≥2.50				
pH	0.731 (0.631–0.831)	**<0.001**	≤7.38	58.49	81.40	79.49	61.40
pO_2_	0.584 (0.469–0.700)	0.153	≤89.50				
pCO_2_	0.502 (0.386–0.619)	0.968	≥39.55				
Lactate	0.614 (0.500–0.727)	**0.049**	≥2.52	43.40	81.40	74.19	53.85
Hemoglobin	0.550 (0.430–0.669)	0.414	≤12.85				
WBC	0.567 (0.451–0.683)	0.258	≤10.41				
Neutrophil	0.522 (0.404–0.640)	0.713	≤22.31				
Platelet	0.618 (0.505–0.731)	**0.041**	≤211.00	54.72	69.77	69.05	55.56
Monocyte	0.534 (0.416–0.652)	0.567	≤0.505				
Lymphocyte	0.570 (0.453–0.687)	0.239	≤1.86				
NLR	0.507 (0.389–0.625)	0.910	≤22.00				
SII	0.594 (0.480–0.708)	0.106	≤2353.05				
PIV	0.550 (0.433–0.667)	0.400	≤1497.85				
ALT	0.543 (0.427–0.658)	0.471	≥41.00				
AST	0.563 (0.447–0.679)	0.286	≥63.00				
Creatinine	0.703 (0.598–0.807)	**<0.001**	≥2.05	49.06	83.72	78.79	57.14
Urea	0.594 (0.477–0.712)	0.116	≥57.50				
Calcium	0.606 (0.490–0.722)	0.074	≥7.95				
Sodium	0.622 (0.509–0.735)	**0.034**	≤139.50	52.83	74.42	71.79	56.14
Potassium	0.517 (0.398–0.636)	0.781	≤4.43				
Procalcitonin	0.570 (0.451–0.689)	0.248	≥0.505				
CRP	0.512 (0.394–0.630)	0.844	≤112.50				
Albumin	0.665 (0.556–0.774)	**0.003**	≤2.95	77.36	53.49	67.21	65.71
INR	0.660 (0.549–0.770)	**0.005**	≥1.315	49.06	79.07	74.29	55.74
PT	0.572 (0.456–0.687)	0.225	≥16.65				
PTT	0.601 (0.488–0.715)	0.081	≤23.65				

Bold values indicate statistical significance (*p* < 0.05). CI: confidence interval; AUC: area under the curve; PPV: positive predictive value; NPV: negative predictive value.

**Table 5:** Cox proportional hazards model for 28-day ICU mortality.

Parameters	Model 1		Model 2		Model 3	
	HR (95% CI)	*p*-Value	HR (95% CI)	*p*-Value	HR (95% CI)	*p*-Value
Age			1.043 (1.001–1.086)	**0.045**		
Sex						
Male			Ref			
Female			1.038 (0.552–1.951)	0.908		
COPD	1.845 (1.067–3.189)	**0.028**	1.504 (0.722–3.133)	0.276		
CRD	1.867 (1.025–3.402)	**0.041**	1.750 (0.748–4.092)	0.197		
Lactate	2.145 (1.243–3.703)	**0.006**	0.764 (0.317–1.839)	0.548		
Platelet	0.500 (0.290–0.861)	**0.012**	0.723 (0.332–1.574)	0.414		
ALT	1.979 (1.112–3.529)	**0.020**	1.665 (0.510–5.438)	0.398		
AST	2.092 (1.175–3.726)	**0.012**	1.643 (0.526–5.135)	0.393		
Creatinine	2.388 (1.383–4.124)	**0.002**	1.167 (0.528–2.578)	0.702	2.683 (1.521–4.731)	**<0.001**
Calcium	2.062 (1.187–3.582)	**0.010**	3.162 (1.473–6.785)	**0.003**	2.312 (1.298–4.118)	**0.004**
Sodium	0.481 (0.278–0.831)	**0.021**	0.859 (0.424–1.741)	0.673		
Procalcitonin	1.953 (1.002–3.805)	**0.049**	1.518 (0.676–3.408)	0.312		
Albumin	0.465 (0.244–0.885)	**0.002**	0.339 (0.158–0.726)	**0.005**		
INR	2.324 (1.349–4.003)	**0.002**	1.329 (0.582–3.038)	0.499		
PT	1.849 (1.027–3.329)	**0041**	1.212 (0.545–2.694)	0.637		
PTT	0.301 (0.162–0.559)	**<0.001**	0.346 (0.150–0.795)	**0.012**	0.396 (0.207–0.758)	**0.005**
VP requirement						
No	Ref		Ref		Ref	
Yes	3.038 (1.561–5.913)	**0.001**	2.007 (0.931–4.328)	0.076	2.225 (1.104–4.484)	**0.025**

Model 1: univariate Cox regression. Model 2: age- and sex-adjusted Cox regression. Model 3: stepwise multivariable Cox regression. Bold values indicate statistical significance (*p* < 0.05). HR: hazard ratio. CI: confidence interval. Ref: reference.

**Table 6:** The 28-day overall survival of ICU patients.

	Survival Estimate (95% CI)	N of Events (%)	Log-Rank $\chi^{2}$	*p*-Value
OS				
All patients	18.00 (15.00–25.00)	53 (55.21)		
PIV			0.191	0.662
Low PIV	18.00 (9.00–25.00)	27 (56.25)		
High PIV	18.00 (13.00–24.00)	26 (54.17)		
COPD			5.148	**0.023**
No	24.00 (15.00–24.00)	31 (46.97)		
Yes	12.00 (7.00–20.00)	22 (73.33)		
Chronic Renal Disease			4.469	**0.034**
No	20.00 (15.00–25.00)	38 (49.35)		
Yes	11.00 (5.00–24.00)	15 (78.95)		

Data are presented as Kaplan–Meier estimates of median survival time with 95% confidence intervals (CIs) and survival probabilities at 28 days. The log-rank test was used to compare survivor functions. OS: overall survival; CI: confidence interval. Bold values indicate statistical significance (*p* < 0.05).

## Data Availability

All data are provided within the article.

## Abbreviations

The following abbreviations are used in this manuscript:

PIV	Pan-immune–inflammation value
SII	Systemic immune–inflammation index
NLR	Neutrophil-to-lymphocyte ratio
APACHE II	Acute Physiology and Chronic Health Evaluation II
SOFA	Sequential Organ Failure Assessment
ICU	Intensive care unit
COPD	Chronic obstructive pulmonary disease
CRD	Chronic renal disease
CVA	Cerebrovascular accident
ALT	Alanine aminotransferase
AST	Aspartate aminotransferase
CVD	Cardiovascular disease
HT	Hypertension
GCS	Glasgow Coma Scale
CRP	C-reactive protein
PT	Prothrombin time
PTT	Partial thromboplastin time
INR	International normalized ratio
ROC	Receiver operating characteristic
AUC	Area under the curve
HR	Hazard ratio
CI	Confidence interval
PPV	Positive predictive value
NPV	Negative predictive value
WBC	White blood cell
DIC	Disseminated intravascular coagulation
OS	Overall survival
